# Body Composition and Physical Health in Sports Practice: An Editorial

**DOI:** 10.3390/ijerph18094534

**Published:** 2021-04-24

**Authors:** Stefania Toselli

**Affiliations:** Department of Biomedical and Neuromotor Sciences, University of Bologna, 40126 Bologna, Italy; stefania.toselli@unibo.it

The assessment of the health status of athletes, at all ages, is an aspect of fundamental importance, and, in recent years, the analysis of body composition has become a fundamental and essential part in its evaluation, such as in the optimization of sports performance.

There are a number of techniques available for estimating body composition, all of which have advantages and disadvantages/limitations [1]. Factors such as the feasibility, cost, technical skill needed, level of accuracy, participant burden, radiation exposure, time taken, validation in an appropriate population, and availability of reference data have to be considered while choosing a suitable method [2].

Body composition can change according to sports practice and more generally by physical activity. It is to be considered that each particular sports discipline requires a specific type of training and activity, and this clearly affects athletes’ body composition; therefore, it is not possible to apply a rigid notion of optimal body composition to every sport [3]. Strategies to achieve the best possible physique for each individual athlete and sports discipline form part of a comprehensive approach to maximize the performance of the athlete. For this purpose, recent studies conducted on athletes have highlighted the need of reference data for assessing the body composition of athletes of different sports, and with or without physical impairments [4,5]. In the absence of appropriate references for each sport category, researchers continue to use reference values from the general healthy population [6]. 

Monitoring and assessing body composition are important, since an adequate physique is a determinant of the athletic performance, success and health of athletes [7]. Attention should be given to preserving the long-term performance and health of the athlete, since physical stress during training and competitions may lead to body composition alterations, which can be detrimental to athletes. This can be achieved by carefully monitoring body composition parameters (fat mass, fat-free mass, hydration status and bone health), understanding the optimal physique for a given athlete, and avoiding potentially harmful practices that may lead to excessively rapid and/or extensive changes in body composition [3].

Finally, particular attention should be paid to young athletes and to their growth and development. Differences in body composition and performance occur in young athletes because of somatic maturation. Individual differences in biological maturity status and timing affect body size, physical fitness, and athletic aptitude in youths and are central to talent identification and development programs in many sports [8,9]. Fat-free mass (FFM) and total body water (TBW) are higher in early maturing athletes, and as a consequence, they present a lower fat percentage (%FM) and a better performance than late mature athletes [10]. This influences the selection—a particularly common scenario in elite teams is the selection of athletes with greater body dimensions and better physical performance, typical in more mature athletes [11,12].

In this Special Issue, titled “Body Composition and Physical Health in Sports Practice” in *IJERPH* [13], 14 papers were recently published on different topics related to body composition. Four papers are on methodological aspects, three papers provide specific reference values, one paper investigates the influence of maturity status on the anthropometric profile and body composition, and six are on the influence of physical exercise and sport practice on body composition and performances. 

Concerning methodological aspects, one paper analyzes the usefulness of raw bioelectrical impedance (BI) parameters in assessing the water compartments and fluid distribution of athletes [14]. Assessing fluid balance to monitor the hydration status in athletes has received substantial interest for maximizing performance, and simple methods are required to assess water compartments and fluid distribution in athletes. The paper showed that raw BI parameters are useful predictors of total and extracellular pools, cellular hydration and fluid distribution in athletes. Another paper considered the specific bioimpedance vector analysis (specific BIVA) to screen and monitor the total body and regional body composition of middle-aged and elderly subjects and affirmed that it appears to be a suitable technique to evaluate the nutritional status and risk of morbidity in these subjects [15]. Body composition changes in different body districts and regional body composition provide relevant information for this purpose, since they allow a better understanding of the role of physical activity in different conditions, such as sarcopenia and obesity. One paper [16] focuses on the analysis of the differences between the formulas used to estimate fat mass and to establish the existing relationship with the body mass index and sums of skinfolds in kinanthropometry. Finally, one paper provides a new predictive model to determine when excess weight is due to excess fat mass [17]. This method could be useful for sports medicine physicians or athletic trainers as an additional assessment of young athletes during physical examinations.

Of the three papers regarding reference values for athletes, one was focused on the development reference values for male and female athletes using classic bioimpedance vector analysis (BIVA) [18]. The paper shows that vector distributions of endurance, velocity/power, and team-sport athletes differ from the general healthy population, and among themselves, due to their different body composition, and thus proposes different reference values. Another paper underlines the importance of bone health valuation of children and youth, given changes in bone mineral density (BMD) and content (BMC) during the course of growth and maturation [19]. In this paper, a reference for bone mineral density (BMD) and content (BMC) specific to young athletes of both sexes participating in several sports was proposed. This could be useful to understand bone development in young athletes and may inform training practices, leading to success in sport, facilitating the diagnosis of structural abnormalities in bone, and contributing to the prevention of skeletal injuries. The third paper provides reference scores on the anthropometric measures and body composition of international-level cerebral palsy (CP) para-footballers that can help sports coaches and physical trainers in the selection process, or to monitor the evolution of players’ body composition [20]. Using these reference scores, coaches could monitor and guide their athletes’ training to achieve the reference values of elite para-footballers. 

A single paper investigates the influence of maturity status on anthropometric profile and body composition and confirms that the majority of the examined variables follow the physiological trend occurring during puberty [21]. The paper underlines the importance of monitoring the anthropometric profile and body composition of children and adolescents participating in sport, as an essential aspect to consider evaluating possible risk conditions and adopting effective countermeasures.

Six papers analyze the influence of physical exercise and sport practice on body composition and performance [22]. One paper focuses on the determination of the effects of dehydration on metabolic and neuromuscular functionality performance during a cycling exercise, pointing out that athletes exercising in a dehydrated state significantly decreased physical performances. This paper underlines the importance of following strategies to maintain a good hydration status during exercise and confirms the ability of BIVA to assess body fluid changes even in sports practice. Another paper details the effectiveness of physical activity interventions in obese subjects, comparing the effects of different weekly resistance training frequencies performed over a 24-week exercise program on phase angle (PA) and handgrip strength (HS) [23]. The paper pointed out that physical exercise performed three times a week promotes better adaptations in PA and HS when compared with the same program performed once a week in obese women. One paper demonstrates that the maximum oxygen uptake (VO2max) calculated per skeletal muscle mass (SMM) is not more useful than conventional VO2max measures to track longitudinal changes in competitive athletes, but it allows one to better distinguish between groups or individuals differing in training status [24]. The focus of another paper was on the examination of the effects of an exhaustive exercise on executive functions, since the effects of acute physical exercise on the cognitive performances of an adult individual are still under discussion [25]. The paper underlines the possibility that high levels of blood lactate induced by exhaustive exercise could adversely affect the executive functions, and stressed that, even with a reduction in quantity, the influences remain present even in older people. One paper refers to changes in estimated body composition prior to a season in elite female and male Polish handball players during a five-week preseason training camp and to sexual dimorphism in response to intensive preseason training [26]. Finally, one paper regards the determination of the dietary acid-base balance in competitive Lithuanian high-performance athletes, and the evaluation of the effect of the diets of athletes on NEAP (net endogenous acid production), muscle mass and body mineral content during a four-year Olympic cycle [27]. The paper showed that, regardless of the type of sport, the diets of Lithuanian high-performance athletes do not meet their requirements, underlying that the training of athletes in different sports needs to be very individualized in terms of the mesocycling of sports goals, including changes in the body composition, giving priority to the formation of eating habits.

In conclusion, the co-editor was satisfied with gathering 14 papers by leading international experts in nutritional status, body composition and exercise. This Special Issue demonstrates the importance of monitoring the health status of the athlete and how body composition assessment represents a fundamental and effective tool to meet this goal.
